# Unexpectedly High Beta-Diversity of Root-Associated Fungal Communities in the Bolivian Andes

**DOI:** 10.3389/fmicb.2016.01377

**Published:** 2016-08-31

**Authors:** Christopher J. Barnes, Carla Maldonado, Tobias G. Frøslev, Alexandre Antonelli, Nina Rønsted

**Affiliations:** ^1^Nina Rønsted Lab, Natural History Museum of Denmark, University of CopenhagenCopenhagen, Denmark; ^2^Herbario Nacional de Bolivia, Universidad Mayor de San AndresLa Paz, Bolivia; ^3^Centre for GeoGenetics, Natural History Museum of Denmark, University of CopenhagenCopenhagen, Denmark; ^4^Department of Biological and Environmental Sciences, University of GothenburgGothenburg, Sweden; ^5^Gothenburg Botanical GardenGothenburg, Sweden

**Keywords:** root-associated fungi, soil fungi, *Cinchona calisaya*, fungal spatial-scaling, beta-diversity

## Abstract

Bolivia is one of the most biologically diverse countries on the planet. Between the Andes and the Amazon drainage basin spans the Yungas, a vast forested region shown to be extremely species rich in macro-organisms. However, it remains unclear whether this high diversity is also reflected in microbial diversity. Here we assess the genetic, taxonomic and functional diversity of root-associated fungi surrounding *Cinchona calisaya* trees, a typical element of the intermediate altitudes of the Bolivian Yungas. We determine the relative effects of edaphic properties, climate, and geography in regulating fungal community assembly. We show that α-diversity for these fungal communities was similar to temperate and arid ecosystems, averaging 90.1 operational taxonomic units (OTUs) per sample, with reads predominantly assigned to the Ascomycota phylum and with a saprotrophic lifestyle. ß-diversity was calculated as the distance-decay rate, and in contrast to α-diversity, was exceptionally high with a rate of −0.407. Soil properties (pH and P) principally regulated fungal community assembly in an analogous manner to temperate environments, with pH and phosphorus explaining 7.8 and 7.2% of community variation respectively. Surprisingly, altitude does not influence community formation, and there is limited evidence that climate (precipitation and temperature) play a role. Our results suggest that sampling should be performed over a wide geographical and environmental range in order to capture the full root-associated fungal diversity in subtropical regions. This study sheds further light on the diversity and distribution of the world's “hidden biodiversity.”

## Introduction

Bolivia is one of the most species rich countries in the world for macro-organisms, whilst its biodiversity is one of the most poorly cataloged (Acebey et al., 2007). Most of this diversity is concentrated to the intermediate zones between the Andean mountains and the Amazonian rainforest, including the Yungas region that extends through most of Peru, Bolivia, and northern Argentina and has been shown to be exceptionally biodiverse (Ibisch, 2001).

Fungi are important within many terrestrial ecosystems, interacting with virtually all plant surfaces and form a vast array of interactions ranging from the beneficial to the pathogenic (Berendsen et al., 2012; Jin et al., 2013), with many thousands of species potentially associating with single trees (Buée et al., 2009; Cordier et al., 2012). Recent studies have suggested that fungal diversity is highest within the tropics (Arnold and Lutzoni, 2007; Tedersoo et al., 2014), whilst subtropical mountain areas have been studied considerably less than temperate and tropical regions. Hence, it remains unclear whether the rich biodiversity in macro-organisms documented for the Yungas is matched by that of microorganisms. With increasing human populations and cultivation, these once pristine environments are being transformed at a rapid rate (Killeen et al., 2008), with the anthropogenic effects of habitat fragmentation and isolation already demonstrated on species richness and composition (Watling and Donnelly, 2008). Given the predicted economical and societal value of the biodiversity within the region (Muller, 2004; Acebey et al., 2007; Suryanarayanan et al., 2009), the race is on to catalog fungal biodiversity and understand ecosystem functioning if effective conservation strategies are to be developed (Scheffers et al., 2012; Costello et al., 2013).

To improve our knowledge on the fungal diversity in subtropical regions in general, and the Yungas in particular, here we investigate the root-associated fungal communities surrounding *Cinchona calisaya* trees. *C. calisaya* is a member of the Rubiaceae family, found within the subtropical mountain zones of the Bolivian and Peruvian Andes (Rusby, 1931). It gained iconic status when it was proven to contain the highest concentrations of quinine and quinine-like alkaloids used for the treatment of malaria, thereby becoming one of the most influential medicines in human history (Achan et al., 2011; Maldonado et al., under review). Whilst it was previously widely distributed, overharvesting for the treatment of fevers has decimated native populations. With the shift in reliance to plantations and the production of synthetic alkaloids, demand for the bark from wild samples has diminished, leaving isolated clusters or individual *C. calisaya* specimens (Kaufman and Ruveda, 2005). The Yungas mountain forest is today the location of a significant proportion of the remaining endogenous *C. calisaya* populations. Consequently the distribution of *C. calisaya* has been studied considerably, and serves a good marker of the traditional Yungas forested region.

Within the Yungas specifically, diverse soil fungal communities have been shown (Geml et al., 2014), whilst a number of different fungal interactions have been shown with *Calisaya* species specifically. Symbiotrophs receive nutrients by exchanging resources with host cells (Nguyen et al., 2016). *Cinchona* species can form associations with symbiotrophic arbuscular mycorrhizal fungi (AMF) within the roots (Schmidt and Scow, 1986) that exchange nutrients from their host for phosphorus and nitrogen scavenged from soil (Neumann and George, 2004). Other potential symbiotrophs include the endophytic fungi that are present within the roots, leaves and stems across higher plant species, and are defined as fungi that exist without causing apparent harm to the host (Petrini, 1991). The potential importance of endophytes has been highlighted specifically within *C. calisaya* (synonym: *C. ledgeriana*), with 21 different endophytic species (from genera including *Xylaria, Diaporthe*, and *Penicillum*) isolated from trees and shown to produce and degrade quinine and quinine-like alkaloids *in vitro* (Shibuya et al., 2003; Maehara et al., 2011, 2012). Meanwhile, the fungal pathogens (fungi that receive nutrients by harming host cells; Nguyen et al., 2016) are some of the most pressing threats to plant production, and supply lines of alkaloids have been comprised by fungal attacks of *Cinchona* plantations (Sawada, 1936). The fungal threats to *C. calisaya* growing naturally within the Yungas region are however considerably less well studied.

Given this importance, the composition and regulation of root-associated fungi has been extensively investigated, showing considerable variation both temporally and spatially (Queloz et al., 2005; Pereira e Silva et al., 2012). These studies have however predominantly been performed within grasslands, agricultural systems, and temperate forests, with very few studies performed within the subtropical mountain regions in which *C. calisaya* is found.

Understanding spatial scaling of microbial communities in mountainous areas is particularly challenging, as there are large gradients in temperature and precipitation associated with changing altitude, and these can occur over relatively short geographical distances (Nekola and White, 1999). Differences in rainfall and temperature have also been shown to affect the composition of root-associated fungi (Dumbrell et al., 2011; Hawkes et al., 2011) and a temperature gradient associated with increasing altitude was shown to influence microbial assembly (Meier et al., 2010). However, these altitudinal gradients across mountains are some of the most studied models within the ecology of macro-organisms, usually producing either linear decline in diversity with altitude or hump-shaped unimodal relationship with altitude (Hillebrand, 2004). Despite shifts in the abundance and composition of soil fungal communities have been observed associated within changing altitude (Bahram et al., 2012; Devi et al., 2012; Geml et al., 2014), it remains unknown whether this pattern mirrors that of macro-organisms.

The effects of dispersal limitation and increasing geographical separation on microbial community composition has been an issue of some contention for microbial ecologists, however there is a growing body of evidence suggesting soil fungi suffer from dispersal limitation at the local (Lilleskov et al., 2004; Barnes et al., 2016), regional (Peay et al., 2007; Põlme et al., 2013), and global scales (Green et al., 2004; Kivlin et al., 2011). Rocks, ravines, roads, and settlements all serve as potential barriers to mixing within the region and high rates of ß-diversity observed for macro-organisms within mountain regions is often attributed to these (Graham and Fine, 2008). A previous study of general soil fungi within the Yungas demonstrated high ß-diversity (Geml et al., 2014), however this also spanned three different forest ecosystems, with considerable changes in the community composition of aboveground biomass. Given microbes ability to reproduce rapidly, asexually, and long-range dispersal mechanisms (Martiny et al., 2011), it remains unclear whether microbial communities suffer the similar effects of dispersal limitation a macro-organisms.

Changing edaphic properties are some of the most influential in determining soil fungal community assemblages. Soil pH has a near ubiquitous effect on microbial communities (Coughlan et al., 2000; Griffiths et al., 2011; Hazard et al., 2013), and has been shown to be a key determinant of the community assembly within mountainous regions (Scattolin et al., 2007; Shen et al., 2013). Other macronutrients such as P, K, and Ca (Gosling et al., 2013; Põlme et al., 2013) have also been suggested to influence fungal community assembly.

In summary, environmental, climatic factors, and geographic separation have been shown to affect the diversity and composition of root-associated fungi. However, the relative effects of each is highly variable and ecosystem specific, and very few studies have investigated fungal diversity in subtropical mountain systems. Using *C. calisaya* as an indicator species for the natural distribution of the hyperdiverse Yungas forests, we (1) categorize the root-associated fungal diversity and (2) determine the role of geographical separation, edaphic properties and climatic variation in regulating the root-associated fungi within the Bolivian Andes.

## Materials and methods

### Sampling sites and sample collection

**Figure 1:**
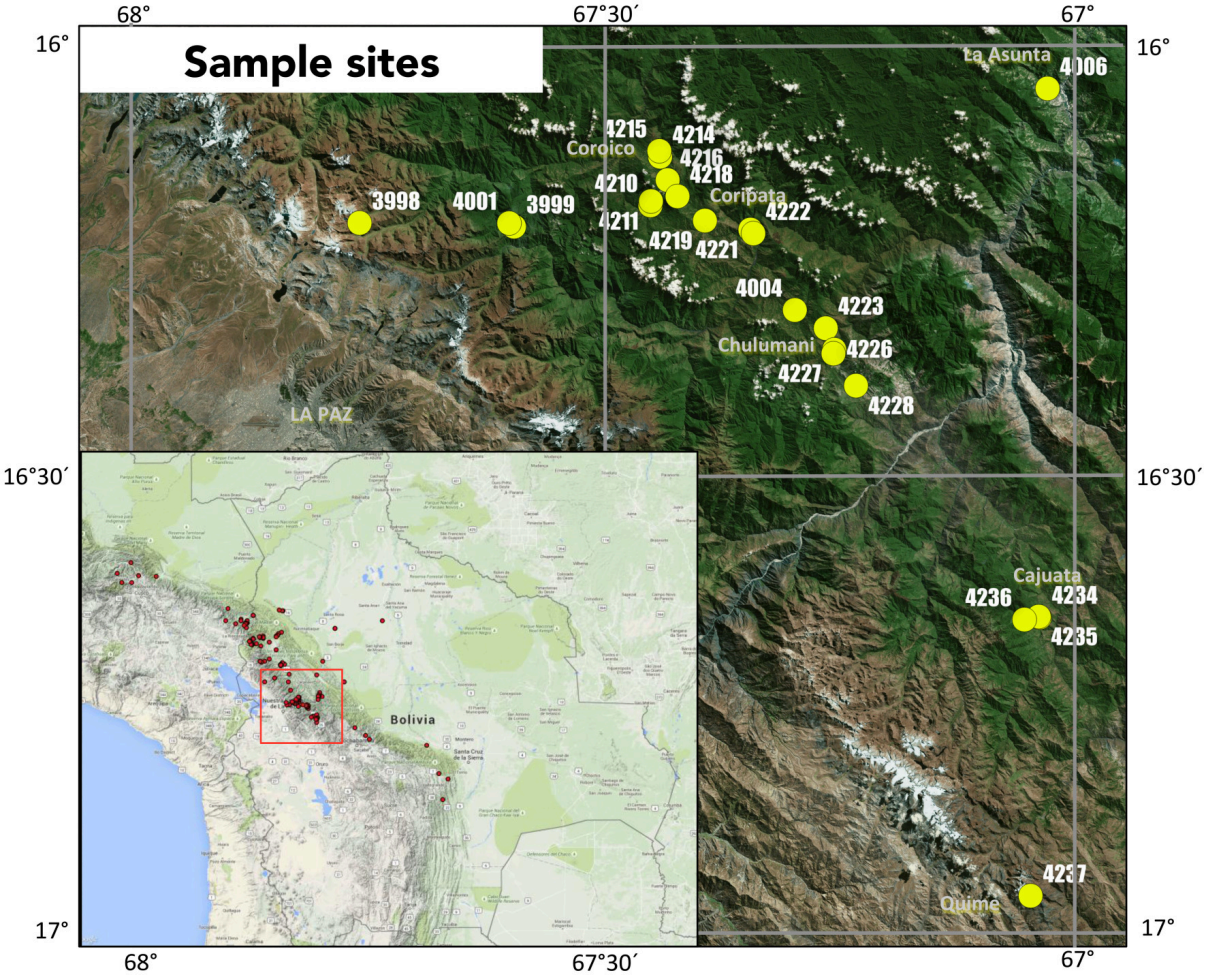
**Map of collection sites and sample names of *Cinchona calisaya*, with the red box indicating the region in the Central Andes of Bolivia where sampling was performed within Bolivia and red dots indicate the full known distribution of *C. calisaya***.

Sampling was performed in the first weeks of October 2012 and October 2014 within the Yungas region of La Paz, Bolivia (Figure 1). In total 21 sites were sampled for this study, with five collected in 2012 and 16 collected in 2014. At each sampling location, three soil samples were taken at a 0.5 m radius around a central *C. calisaya* tree, with approximately 100 g of soil taken from the uppermost 15 cm of the soil profile. Soil samples were homogenized by gloved hand and taken from the field, then air-dried for 3 weeks in partially open sterile bags, and finally stored in opaque sealed containers at room temperature. Lignified roots were manually extracted from dried soil based on morphology using forceps. Vouchers were taken for each associated tree specimen, with duplicates deposited in the Bolivian National Herbarium, La Paz, Bolivia (LPB), and the Natural History Museum of Denmark, University of Copenhagen, Denmark (C).

### Characterization of edaphic and climatic variables

Dried soil was sieved to under 2 mm and further homogenized by hand, before 35 g of the resultant powder was pooled together from the three soil subsamples. Edaphic properties including pH, humus, %C, %N, Mg (mg/kg), P (mg/kg), and K (mg/kg) were determined by Eurofins (Denmark) as part of a previous study exploring chemical diversity of *C. calisaya* trees (Maldonado et al. under review). Methods were outlined by the Danish Agrifish Agency under the Ministry of Environment and Food of Denmark (Sørensen, 1994). Humus was determined in a CO_2_ free environment by an automated C/N analyser (LECO Tru Mac N; Leco, Michigan, USA) in conjunction with a muffle furnace (Nabertherm, Germany). Nitrogen was extracted as ammoniacal nitrogen (NH4-N) by distillation and titration after the destruction of organic matter with sulfuric acid and quantified using a LECO Tru Mac N. Olsen's P was determined spectrophotometrically as per the standard operating procedure (Foss, FIAstar 5000 Analyzer). Flame photometry and spectrophotometry were used to determine K and Mg respectively using an (AA) ICP-OES (iCAP 6500 Radial, Thermo Scientific, USA) coupled with plasma optical emission spectrometry. Finally, pH was determined in deionized water using a pH meter (ACCUMET AB15, Fischer Scientific, UK) and automated using a custom built robot).

In order to investigate spatial relationships among fungal communities, GPS coordinates and altitude were taken using a handheld GPS (GARMIN *eTrex* H, USA) at each sampling site. Using these coordinates, the climate was characterized by downloading the mean annual temperature and precipitation for each site at 30 s resolution from the WorldClim database (Hijmans et al., 2005).

### Fungal metabarcoding of roots

For the present study, DNA extractions of roots were performed using a PowerSoil DNA isolation kit (MP Biomedicals, Cambridge, UK). For each sample, 500 mg of roots were placed in tubes before the addition of lysis buffers, then underwent three periods of shaking at 30 Hz/s for 30 s using a tissue lyser (TissueLyser II, QIAGEN, Denmark). Extractions were subsequently performed as per manufacturer's instructions.

PCR metabarcoding of the fungal ITS region was performed with ITS1F (Gardes and Bruns, 1993) and glITS7 primers (Ihrmark et al., 2012). In order to increase number of samples multiplexed, internal tags that ranged between 7 and 8 bp long were attached to the forward and reverse primers, with a minimum of 3 bp difference between internal tag sequences. PCRs were performed using 1 μL of DNA extracts, 0.2 μL of AmpliTaq Gold (Applied Biosystems, Forster City, CA), 2.5 μL of x10 buffer, 2.5 μL of 25 mM MgCl_2_ 1 μl of each primer (at 25 mM/μL), 0.2 μL of 25 mM dNTPs (Invitrogen). PCR conditions consisted of: 95°C for 5 min, then 30 cycles of 95°C for 30 s, 56°C for 30 s, 72°C for 30 s, and a final extension of 72°C for 10 min. PCR products underwent purification using a QiaQuick PCR Purification Kit (QIAGEN, Denmark) following manufactures instructions.

PCR products were pooled randomly into three libraries. Size selection and further purification was performed on pooled PCR products using a 2% E-Gel EX with SYBR Gold II, ran on an E-Gel iBase (Life Technologies, Carlsbad, CA, USA), with products of approximately 340 bp selected for. In order to convert samples into Illumina sequencing libraries, a NEBNext DNA Library Prep Master Mix Set for 454 (#E6070, NEB, Ipswich, MA, USA) was used. This was performed as per manufacturers guidelines; however, blunt-end p5/p7 Illumina adapters (Meyer and Kircher, 2010) were substituted in the place of the Roche FLX adapters.

Libraries underwent a second PCR reaction to add Illumina tags. Samples were equilibrated to 25 μM using molecular grade water and PCR reactions contained: 5 μL of blunt-end products with 0.2 μL of AmpliTaq Gold (Applied Biosystems, Forster City, CA), 2.5 μL of x10 buffer, 2.5 μL of 25 mM MgCl_2_, 0.2 μL of 25 mM dNTPs (Invitrogen) and 1 μM of BSA (NEB), 1 μL of primer paired end 1 μL of 10 mM (InPE1.0) forward primer and 1 μM of 10 mM tagged reverse index primer. Cycling conditions were: 95°C for 10 min, then 12 cycles of 95°C for 30 s, 60°C for 30 s, 72°C for 30 s, before undergoing a final extension of 72°C for 7 min. Finally, products were purified using a 1:1 ratio of product to AMPure XP magnetic beads (Beckman Coulter Inc, Denmark) and visualized on a 2100 Bioanalyzer (Agilent Technologies, Santa Clara, CA, USA). Completed libraries were run using 250 base pair paired-end sequencing on the Illumina MiSeq platform across an entire MiSeq flowcell.

### Bioinformatic analyses

Library pools were demultiplexed using a custom script, with only those with absolute matches for both forward and reverse primers tags assigned to samples. Paired ends were joined in QIIME (v1.9) before undergoing denoizing using USEARCH (v8.0), which removed all sequences shorter than 150 bp and those with a likelihood of 1 bp error or greater (Caporaso et al., 2010; Edgar, 2010). Chimeras were removed using the reference based UCHIME chimera checking (v4.2; Edgar et al., 2011), utilizing the UNITE 31.01.16 general FASTA format release (Abarenkov et al., 2010). Adapters were removed using CutAdapt (v1.9.1) before OTUs were picked at the 97% similarity level in QIIME using the UCLUST algorithm (v1.2.22). The ITS2 region was extracted using ITSx (ITS extractor, v1.0.11) for each OTU (Martin, 2011; Bengtsson-Palme et al., 2013) and taxonomy was assigned using BLAST in QIIME against the UNITE fungal ITS database (2016-01-31 release). Additionally, due to a high proportion of poorly taxonomically resolved OTUs, OTUs assigned at the phylum level or higher that were persistent (defined by being present in 5 or greater samples) underwent a second round of taxonomic assignments. These OTUs manually underwent BLAST searches against NCBI nucleotide collection (nr/nt) reference database and a new taxonomy was assigned if sequences were found with 99% or greater similarity. Singletons (ie single unique reads) were removed from the OTU table, and reads subsequently rarefied to the lowest sampling depth, 2000 per sample. Finally, the OTU table was parsed against the FunGuild (v1.0) database to assign putative life strategies to taxonomically defined OTUs (Nguyen et al., 2016).

### Statistical analyses

To test for significant undersampling, total OTU richness (Chao1) was estimated for each sample using QIIME (Chao, 1987). Total OTU richness estimates were compared to observed OTU richness using a paired *t*-test. ß-diversity was assessed in the form of a distance-decay rate (DDR), using the formula S = cD^DDR^, where S is the community similarity value between two samples, c is a constant, D is their geographical distance apart and the exponential is the DDR. Community similarities were calculated as Jaccard similarity values utilizing data on a presence-absence basis (Jaccard, 1901; Real and Vargas, 1996). An Euclidean distance matrix was created from GPS coordinates for determining the geographical distances between paired samples (using the Ape package within *R*; Paradis et al., 2004).

The effects of changing geographical, climatic, and edaphic properties in determining the root-associated fungal community assembly were tested in a number of ways. Initially, the community was visualized using non-metric multidimensional scaling from a non-binary Jaccard similarity matrix. Significance of each individual edaphic and climatic parameters were tested separately against the ordination of the root-associated community using the envfit function. In order to test the effects of geographical separation, a principle coordinates of neighbor matrix (PCNM) was established from GPS coordinates and the first two principal coordinates (PCNM1 and PCNM2) that were analyzed against the root-associated fungal communities, also using the envfit function. The Jaccard community similarity matrix formation, NMDS, PCNM formation, and envfit analyses were all performed using the Vegan package of *R* (Oksanen et al., 2016).

The effects of geographical separation, climatic parameters and edaphic properties were also investigated simultaneously via PERMANOVA. For this analysis, the ADONIS function was used, also in the Vegan package within *R* (Oksanen et al., 2016). As before, PCNM1 and PCNM2 were included as geographical variables. However, due to high autocorrelation between the edaphic properties pH, %N, %C, humus, K (mg/kg), and Mg (mg/kg), only pH and P (mg/kg) (which did not correlate with the other edaphic properties) were included within the PERMANOVA. The effect of collecting over the 2 years (2012 and 2014) was also investigated by including the year of sample collection as a factor, whilst the year of collection also served as the grouping variable. Thus, within the final analysis, the fungal community was analyzed (in presence/absence form) against the pH, P (mg/kg), precipitation (mm/yr), temperature (°C), altitude (m), PCNM1 and PCNM2, whilst, and year of sampling served as a factor and as the grouping variable.

Finally, OTU richness underwent linear modeling with the parameters using the pH, P (mg/kg), precipitation (mm/yr), temperature (°C), altitude (m), year of sampling, and the first principal components produced from the PCNM, using both forward and reverse selection (utilizing the lm function within *R*).

## Results

### Soil and climatic properties

C ranged between 97.5 and 60.5%, whilst nitrogen ranged from 1.21 to 0.07% and humus ranged from between 40.6 and 2.5% (Table 1). The macronutrients Mg and K were relatively abundant within samples, ranging from 88.0 (mg/kg) to 2.0 (mg/kg) and 26.0 (mg/kg) to 3.9 (mg/kg), respectively. Soil conditions were highly acidic, with pH ranging from as low as 4.0 to near neutral 6.1. A strong autocorrelation among all of pH, C, N, humus, Mg, and K (*R*^2^ > ±0.013, *P* > 0.01) was found. P content was very low, ranging from just 1.2 (mg/kg) to < 0.01 (mg/kg) and did not correlate with the other edaphic properties.

**Table 1:** **List of climatic parameters and edaphic properties at each sampling site within the Bolivian Andes**.

**SampleID**	**Temp (°C)**	**Prec (mm)**	**Alt (m)**	**Humus (%)**	***C* (%)**	***N* (%)**	**pH**	***P* (mg/kg)**	***K* (mg/kg)**	**Mg (mg/kg)**
CMG3998	15.33	638	1820	14	85.7	0.45	4.7	0.9	13	9.3
CMG3999	11.55	828	1800	16	83.7	0.53	5.2	0	10	7.4
CMG4001	11.55	828	1800	16.5	84.5	0.5	4.6	0	7.2	2.2
CMG4004	11.55	828	1760	6.3	93.7	0.27	4.9	0	9.5	17
CMG4006	15.66	1810	747	9.9	90.1	0.36	5.4	1	25	58
CMG4210	10.87	1180	1914	26	74.1	0.74	4.1	0	26	7.3
CMG4211	10.87	1180	1914	28	72.4	1.01	4	0	23	3.3
CMG4214	13.81	1392	1305	12	88.5	0.63	5	1.2	17	12
CMG4215	13.81	1392	1305	5.8	94.2	0.2	4.9	0	6	5.2
CMG4216	11.09	1210	1910	18	82.3	0.5	4.2	0	14	2
CMG4218	10.27	1202	1900	40	60.5	1.21	4.1	0	20	2.7
CMG4221	13.84	1368	1348	6	94	0.19	5.7	0	20	38
CMG4222	13.84	1368	1134	2.5	97.5	0.07	6.1	0	3.9	44
CMG4223	14.2	1366	1123	6.1	93.9	0.2	4.4	0	3.9	88
CMG4226	13.52	1309	1264	5.1	94.9	0.2	5	0	11	10
CMG4227	13.87	1325	1209	2.9	97.1	0.11	4.7	0	7.5	7.3
CMG4228	11.55	1282	1260	2.8	97.2	0.1	4.9	0	5.1	10
CMG4234	15.03	1142	2092	4.7	95.3	0.29	5.6	0.4	15	34
CMG4235	15.03	1142	2092	6.2	93.8	0.21	5.6	0.5	13	28
CMG4236	15.46	1187	1947	2.8	97.2	0.15	5.1	0	6.8	3.4
CMG4237	17.53	558	2050	3.3	96.7	0.14	5.9	0	7.3	12

Temp, Temperature; Prec, precipitation (mm); Alt, altitude (m).

Climatic parameters also varied between sampling sites (Table 1). As a result of mountainside sampling, altitude varied greatly, between 747 and 2092 m above sea level. Precipitation ranged from 558 to 1810 (mm/yr) and correlated with altitude (*R*^2^ = −0.710, *P* > 0.001). Temperature did not correlate with precipitation and altitude (*R*^2^ = −0.011, *P* = 0.962 and *R*^2^ = −0.136, *P* = 557), but varied between 10.27 and 17.5°C.

### Characterizing the root-associated fungal community

**Figure 2:**
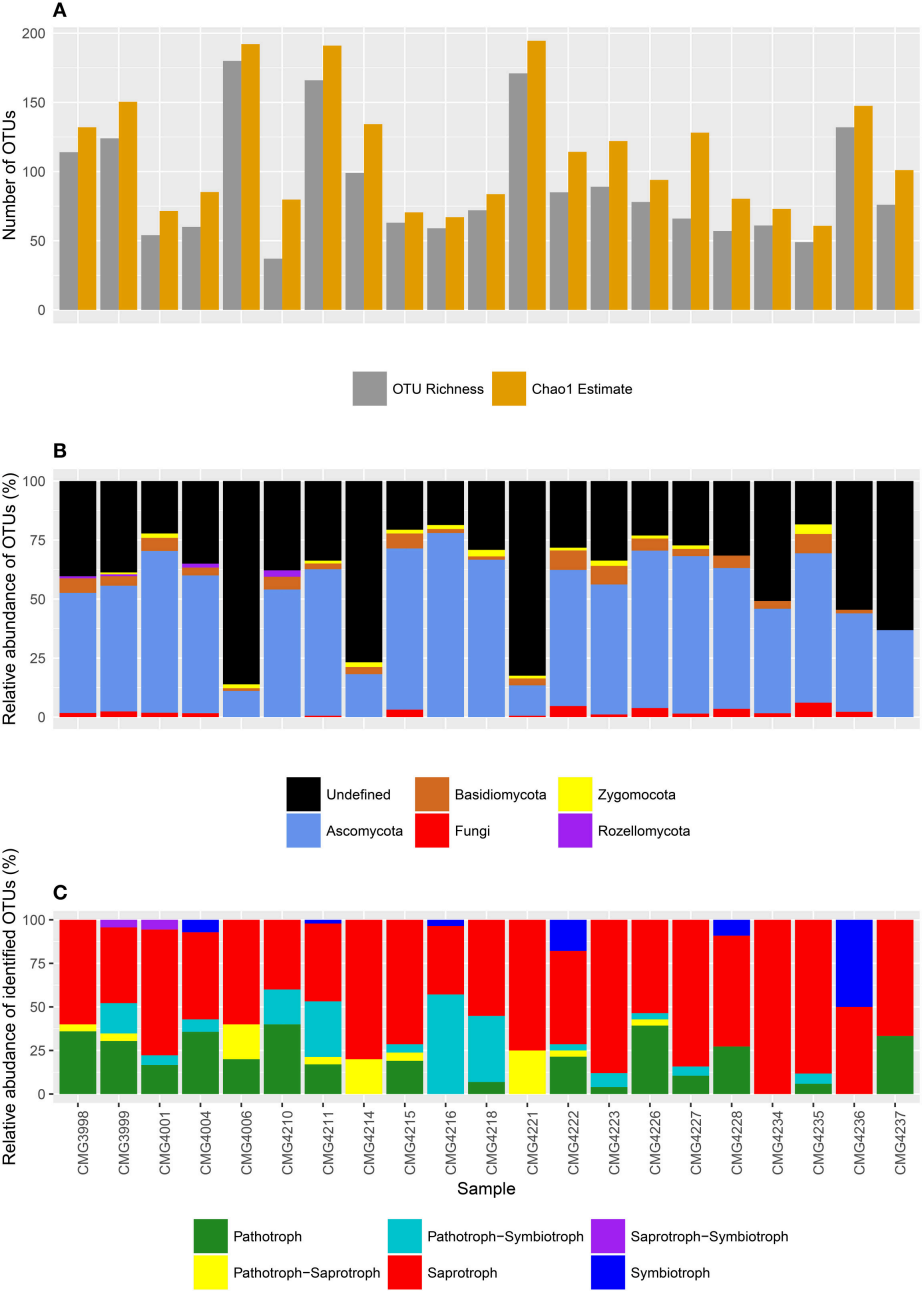
**(A)** OTU richness and Chao1 estimates (predicted total diversity), **(B)** taxonomic composition at the phylum level, and **(C)** composition of life strategies at each sampling site within the Bolivian Andes.

Reads were rarefied to a sampling depth of 2000 per sample, yielding a total of 42, 000 reads over 896 OTUs in total, with a mean read length of 364 bp. OTU richness varied from just 37 to 180 OTUs, with an average of 90.1 observed OTUs per sample (Figure 2A). However, Chao1 estimates indicate a significant undersampling (*t* = −8.105, *P* > 0.001), with an estimated 22.9 OTUs per sampling being missed on average (113.0 OTUs), with estimated total OTU richness ranging from between 60.8 and 194.6 OTUs.

Reads were assigned to 4 fungal phyla, including the Ascomycota, Basidiomycota, Zygomycota, and Rozellomycota (Figure 2B). The Ascomycota were the most abundant, with an average of 42.1 OTUs per sample (ranging between 18 and 103 OTUs), dwarfing the other phyla. By comparison, other phyla were present in low abundance, with reads assigned to an average of just 3.3 Basidiomycota OTUs per sampling site (ranging between 0 and 7 OTUs), 1.0 Zygomycota OTUs (ranging between 0 and 3 OTUs), and only 0.2 Rozellomycota OTUs per sample (ranging between 0 and 1 OTU). There were also a substantial number of poorly assigned OTUs, with an average of 1.4 OTUs assigned to the fungal kingdom only (ranging from 0 to 4) and a further 42.0 OTUs completely taxonomically unassigned (ranging from 9 to 155 OTUs, respectively). Of the “persistent” OTUs (found in 5 or more samples), 38.8% were not defined to phylum level or higher using the UNITE database. This figure dropped to 28.4% of OTUs with the further round of taxonomic assignments by manual BLAST searches.

Using FunGuild, putative life strategies were assigned to OTUs based on taxonomic assignments (Figure 2C). Saprotrophs were the most abundant, with an average of 10.7 OTUs assigned to saprotrophs (ranging from 2 to 22), and a further 0.48 OTUs (range from 0 to 2 OTUs) as pathotroph-saprotrophs and 0.01 OTUs were saprotroph-symbiotrophs (range of 0 and 1 OTUs). There was an average of 3.2 OTUs assigned to pathotrophs (ranging between 0 and 11.0 OTUs), whilst pathotroph-symbiotrophs accounted for a further 2.7 OTUs (ranging between 0 and 16 OTUs). Symbiotrophs were the least abundant life strategy, with reads assigned to symbiotrophic OTUs accounting for 0.5 OTUs per sample site and ranging between 0 and 5 OTUs.

**Figure 3:**
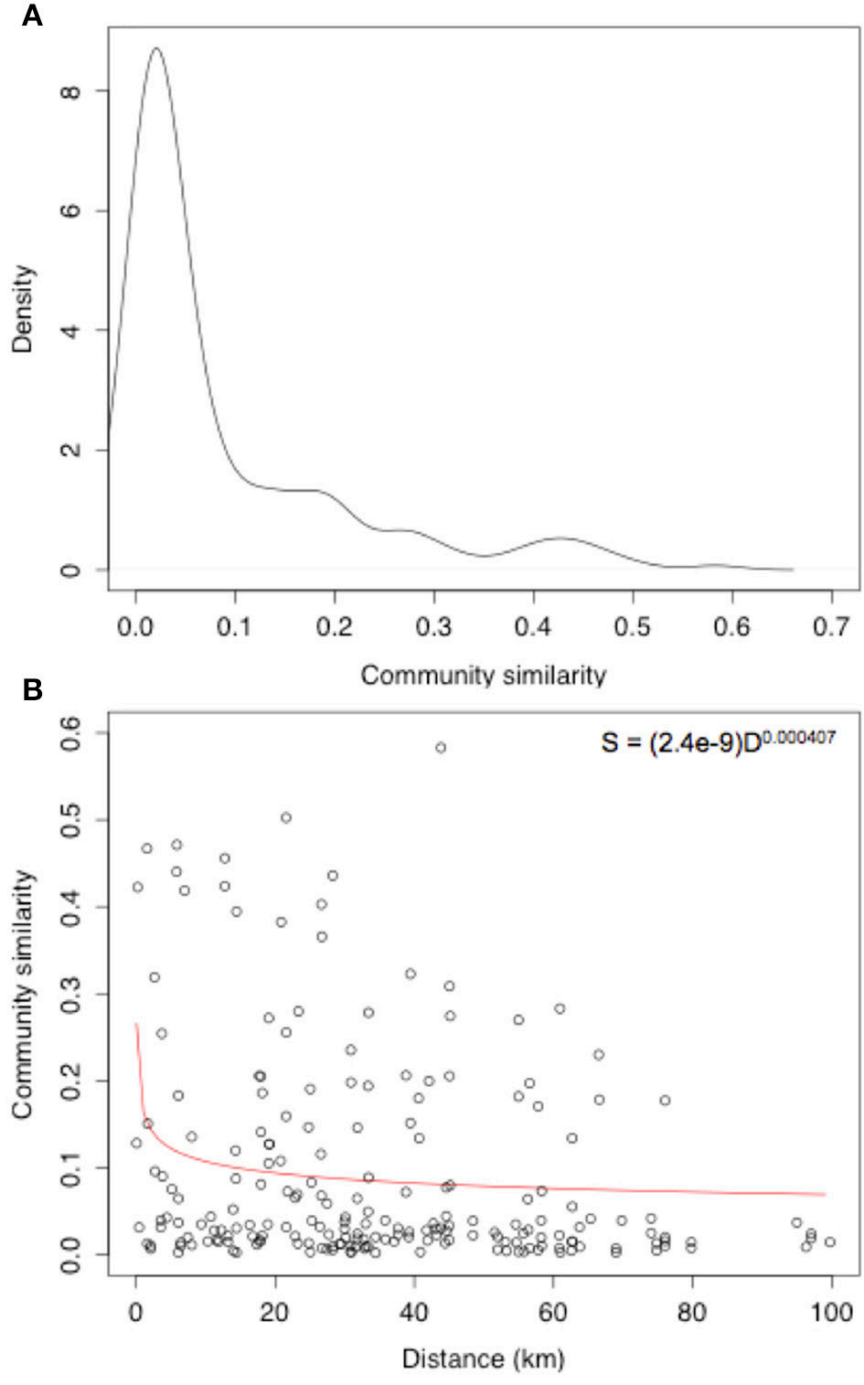
**(A)** Community similarity density and **(B)** distance-decay rate of root-associated fungi of the Bolivian Andes.

The distribution of Jaccard similarity measures for the root-associated fungal community was centered at extremely low values, with a median community similarity between samples of just 0.032 and a mean of 0.091 (Figure 3A). A DDR was also calculated across the sites as a measure of ß-diversity, with a DDR of -0.407 m (Figure 3B).

There were 29 OTUs distributed over a third of all samples (Table 2). There were a number of highly persistent OTUs assigned to the Ascomycota including; 2 *Chaetothyriales* sp., *Nectriaceae* sp., *Talaromyces* sp., and a *Tremellomycetes* sp. which were present in over two thirds of samples. Whilst no other phyla were represented within the highly persistent OTUs, there was a further 8 unassigned highly persistent OTUs. Where possible, these were putatively assigned to life strategies, with 8 saprotrophs, 2 pathotrophs, and 5 pathotroph-symbiotrophs represented, whilst 6 OTUs could not be taxonomically assigned to beyond the phylum level.

**Table 2:** **List of the taxonomic assignment, putative life strategy, and persistence of root-associated fungal OTUs from the Bolivian Andes (occurring in over 30% of samples)**.

**Taxonomy**	**Trophic mode**	**Persistence**
*Chaetothyriales* sp.1	–	18
*Nectriaceae* sp.	–	18
*Chaetothyriales* sp.2	–	17
*Talaromyces* sp.	Saprotroph	17
*Tremellomycetes* sp.	–	15
*Oidiodendron* sp.	Pathotroph-Symbiotroph	13
*Umbelopsis* sp.	Saprotroph	12
*Ascomycota* sp.	–	10
*Chaetomium* sp.	Saprotroph	9
*Clonostachys rosea*	Pathotroph	9
*Leohumicola* sp.	Saprotroph	9
*Aspergillus* sp.	Saprotroph	8
*Chaetomiaceae* sp.	–	8
*Cladosporium* sp.	–	8
*Coniochaetales* sp.	–	8
*Pestalotiopsis* sp.	Pathotroph	8
*Eurotiomycetes* sp.	-	8
Talaromyces verruculosus	Saprotroph	8
*Unassigned OTU1*	–	8
*Dothideomycetes* sp.	–	7
*Helotiales* sp.	–	7
*Hyphodontia* sp.	Saprotroph	7
*Pleosporales* sp.	–	7
*Sordariales* sp.	–	7
*Brycekendrickomyces acaciae*	Saprotroph	7
*Oidiodendron* sp.	Pathotroph-Symbiotroph	7
*Unassigned OTU2*	–	7
*Unassigned OTU3*	–	7
*Unassigned OTU4*	–	7

### Assessing the environmental and geographical regulation of root-associated fungi

**Figure 4:**
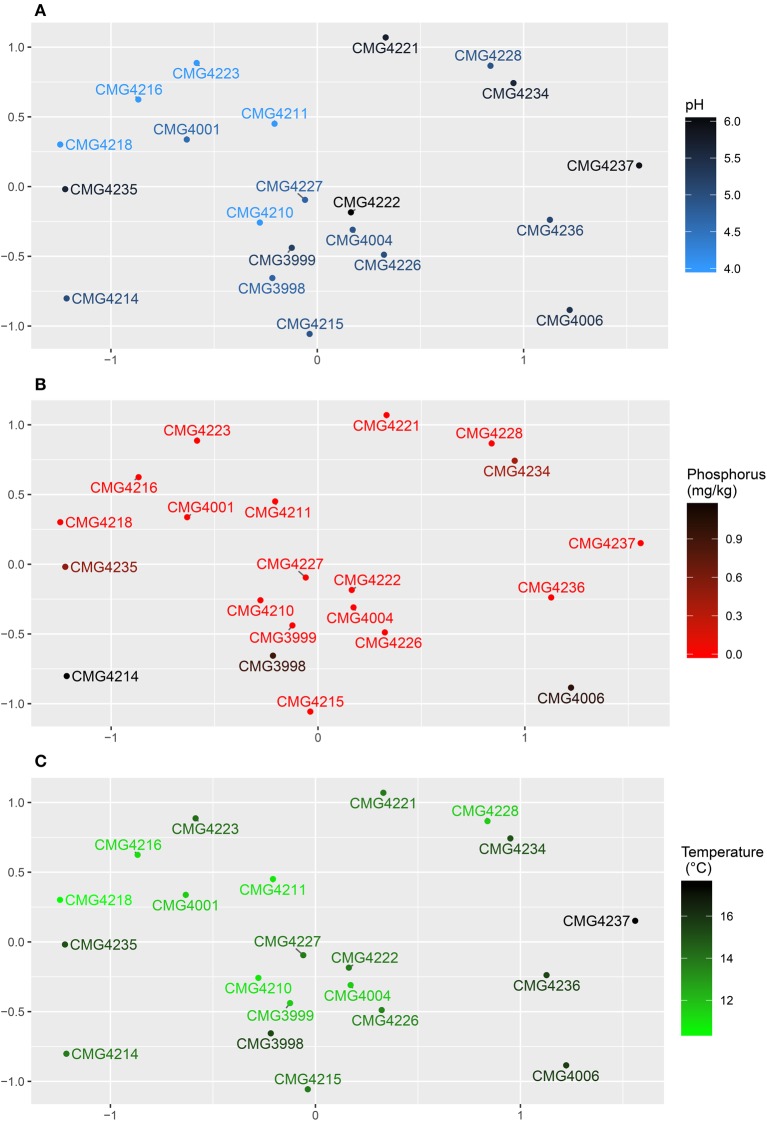
**Non-metric multidimensional scaling plots for root-associated fungi of the Bolivian Andes**. Points represent the community within the ordination. Sample labels are given, with color representing: **(A)** pH, **(B)** P (mg/kg), and **(C)** Temperature (°C).

Fungal community data was visualized using non-metric multidimensional scaling and individual parameters correlated against the community using the envfit function. The autocorrelated cluster of soil pH (*R*^2^ = 0.3758, *P* = 0.016; Figure 4A), %C (*R*^2^ = 0.2846, *P* = 0.034), %N (*R*^2^ = 02776, *P* = 0.043), humus (*R*^2^ = 0.2924, *P* = 0.031), and K (mg/kg) (*R*^2^ = 0.2382, *P* = 0.045) all correlated with the community whilst Mg (mg/kg) did not. P (mg/kg) also correlated with the fungal community composition (*R*^2^ = 0.3128, *P* = 0.035; Figure 4B). Temperature (°C) was the only climatic variable to correlate with the community (*R*^2^ = 0.2986, *P* = 0.048; Figure 4C), whilst geographic effects were also linked to the community in the form of PCNM1 (*R*^2^ = 0.2995, *P* = 0.036).

PERMANOVA was performed to simultaneously test the relative effects of edaphic, climatic and geography on the root-associated fungal community (Table 3), revealing significant effects of soil pH (*P* = 0.013), phosphorus content (*P* = 0.034) and precipitation (*P* = 0.018), explaining 7.8, 7.2, and 7.6% of community variation, respectively. There were no effect of geography, altitude and temperature.

**Table 3:** **ADONIS analysis showing the correlation between climatic, soil, and geographical parameters against the root-associated fungal community composition of the Bolivian Andes**.

**Parameter**	**Variance explained (%)**	***P*-value**
pH	7.796	0.013
Phosphorus (mg/kg)	7.16	0.034
Precipitation (mm/yr)	7.641	0.018
PCNM1	0	0.316
PCNM2	0	0.983
Temperature (°C)	0	0.753
Year of collection	0	0.97
Total	22.597	

Finally, linear modeling was performed to link environmental and geographical parameters to OTU richness. However, both forward and reverse stepwise regression found no significant effect of these parameters on OTU richness.

## Discussion

We show that the root-associated fungal communities within the former Yungas forests are relatively diverse, with potentially many fungi likely to be new to science. However, at an average of just 113.0 OTUs per sample site, α-diversity was not high even when compared to other studies of root-associated fungi of single plant species within temperate forests (Buée et al., 2009), alpine (Bjorbækmo et al., 2010), and artic systems (Blaalid et al., 2012), and diversity within the region was primarily driven by ß-diversity. The Ascomycota dominated the community, with saprotrophic and pathotrophic life strategies being the most abundant. There were also 2 OTUs assigned to the recently described Rozellomycota phylum (James and Berbee, 2012) that were present in low persistence. There was also a substantial proportion of OTUs that could not be taxonomically assigned to even the fungal kingdom. Within metabarcoding studies, a proportion of OTUs will invariably be poorly taxonomically assigned. However, at approximately 28.4%, the proportion of poorly taxonomic defined OTUs within this study was considerably higher than in studies within other environments (Tedersoo et al., 2014), suggesting that like macro-organisms, the microorganisms of Bolivia are poorly characterized.

Community level studies with detailed taxonomic compositions have only been possible with recently developed sequencing techniques, thus studies of the composition of fungal life histories has rarely been performed. OTUs assigned to plant pathogens were shown to be particularly diverse within the system. These fungi usually attack a limited number of phylogenetically related hosts (Gilbert and Webb, 2007), thus their relative high diversity might be in response to the hyperdiverse aboveground communities of the Yungas region, which presents many potential niches for pathogens to diversify within. Pathogenic OTUs included *Fusarium* and *Volutella* species that have extremely broad ranges of hosts (Rowe, 1980; Shi and Hsiang, 2014), and could be antagonizing the roots of *C. calisaya*. Meanwhile global saprotroph diversity was shown to positively correlate with mean annual precipitation (Tedersoo et al., 2014), which is notably high within the region. Furthermore, the high quantities of soil C suggest an abundance of organic matter for which the observed diverse saprotrophic community could be maintained. There were also very few OTUs assigned to symbiotrophs within the system, with ericoid mycorrhizal the most abundant with a further root endophyte observed also. Ericoid mycorrhizal associations can only occur with Ericaceous hosts that are common within the neo-tropics, but does not include *C. calisaya* (Cairney and Meharg, 2003).

Studies of macro-organisms within the region found that even in species rich areas, only a relatively low proportion of total richness is present within sampling locations (Kessler, 2001; Herzog et al., 2005). With only 29 OTUs present in over a third of samples, and the mean OTU persistence being just 10.0% of samples, this pattern appears also likely for the root-associated fungi of the Bolivian Andes. Pairwise similarity values for the root-associated fungal community were also very low, with a mean similarity of just 0.091 between samples, suggesting stochastic rather than environmental processes regulate community similarities. This in turn is reflected by the extremely high ß-diversity compared to other studies of soil fungal communities at the local (Barnes et al., 2016), regional (Hazard et al., 2013), and global levels (Green et al., 2004).

Community assembly of the root-associated fungal community was predominantly driven by edaphic properties, rather than climatic parameters and geographical separation. Soil pH explained 7.8% of community variation despite being acidic in all samples, which is in agreement with the general consensus within microbial ecology (Griffiths et al., 2004; An et al., 2008; Tedersoo et al., 2014), whilst the importance of soil pH has been previously demonstrated across altitudinal gradients (Scattolin et al., 2007; Shen et al., 2013). Although pH correlated with a number of edaphic properties, including K, Mg, C, N, and humus, pH consistently explained the highest percentage of variation within the PERMANOVA analysis and had the strongest correlation with the fungal ordination using the envfit analyses. These results coupled with the near ubiquitous effect of pH on microbial communities, suggest that pH was primarily driving variation within the community despite autocorrelating with a number of other edaphic properties.

Soil P (mg/kg) however did not correlate with pH and the other edaphic properties, but significantly explained a further 7.2% of variation within the root-associated fungal community. Within the tropics, soils are often very old, thus soil N is relatively high while P is low (Vitousek, 1984). There was variation in soil N (between 1.21 and 0.07%), but P content was consistently low, ranging from just 1.2 (mg/kg) to < 0.01 (mg/kg) and at these concentrations impact upon the aboveground biomass (Lambers et al., 2008). No AMF and very few symbiotrophs taxa were detected within the communities. This is surprising, given their ability to enhance plant P uptake (Neumann and George, 2004), previously demonstrated high abundance and diversity within the Yungas forests of Argentina (Becerra et al., 2011), and ability to colonize *Cinchona* species in high numbers (Schmidt and Scow, 1986).

Whilst Geml et al. (2014) found altitude principally determines fungal community within the Andes, possibly through changing of precipitation and rainfall, here we did not find altitudinal effect and limited support of changing climatic parameters influencing fungal communities, despite also sampling over similar altitudinal ranges (400–2160 m in (Geml et al., 2014), 747–2092 m within this study). Altitudinal associated shifts in the aboveground biomass are well documented (Hillebrand, 2004), and a major difference between this study and Geml et al. (2014), and also other studies that have observed altitudinal effects on soil fungal communities (Bahram et al., 2012; Devi et al., 2012; Gai et al., 2012), is that attempts were made to limit changes within the composition of the aboveground biomass (as sampling within this study limited to around *C. calisaya* species only). When changes in the aboveground communities are limited, the effects of altitude on microbial communities becomes less clear (Scattolin et al., 2007; Nouhra et al., 2012), thus it remains unknown whether many of the observed altitudinal shifts in fungal assemblages are direct responses to changing altitude or as an indirect effect of changes in the aboveground biomass.

There was limited evidence of geographical separation influencing the root-associated fungal composition, with PCNM1 correlating the community using the envfit approach but not within the PERMANOVA. ß-diversity however far exceeded that found in other regions, even those with comparable variation in edaphic and climatic properties (Green et al., 2004; An et al., 2008; Barnes et al., 2016). Across the Yungas there are many steep edges and ravines that could serve as natural barriers to dispersal between microbial populations (Killeen et al., 2008). Thus, there may not be a linear relationship between community similarity and increasing geographical distance, but rather a more cryptic pattern of spatial scaling, with fungal communities varying disproportionately over these barriers. Whilst there is a clear effect on the composition of the aboveground biomass by harsh mountain topology (Nekola and White, 1999), further studies are required to determine whether they can similarly influence microorganisms.

Finally, it should be noted that sampling was only in areas in which *C. calisaya* can grow, whilst rarely performed in undisturbed environments. Additionally, whilst sampling storage time did not have a significant effect on the community composition and OTU richness, air-drying of samples has been previously demonstrated to lead to decreased measurements of total fungal richness using molecular techniques (Clark and Hirsch, 2008). Therefore, total root-associated fungal richness within the region was not assessed whilst sample preparation may of reduced observed α-diversity, which is likely to be considerably higher, as previously found for general soil fungi by Geml et al. (2014).

## Conclusions

The high percentage of uncharacterized fungi found in this study, coupled with very high ß-diversity, suggest that cataloging the full fungal biodiversity in the Yungas could yield an upwards revision of estimates of the total number of global fungal diversity (Tedersoo and Nara, 2010; Hawksworth, 2012). Thus, there is an urgent need for α-taxonomic work and establishing reference DNA databases for fungal diversity in tropical and subtropical regions in general, and the Andes in particular. Furthermore, results suggest that whilst α-diversity is not exceptional, a high turnover of species contributes substantially to the total fungal diversity within the region, and this might be driven by dispersal limitation. Community regulation by edaphic properties such as pH and P appears to be analogous to that of well-studied temperate environments. Future studies would therefore benefit in characterizing fungal communities over differing geographical scales, and between potential barriers to mixing, in order to optimize our understanding of root-associated fungal communities in mountainous regions.

## Author contributions

CB produced data, performed data analysis and wrote the manuscript. CM collected samples and assisted in data generation. TF designed primers essential for fungal community data production, assisted in bioinformatical analysis and also manuscript writing. AA assisted in collecting samples, data interpretation and manuscript writing. NR raised funds, collected samples, and assisted in manuscript writing.

### Conflict of interest statement

The authors declare that the research was conducted in the absence of any commercial or financial relationships that could be construed as a potential conflict of interest.
